# The electrochemical-step asymmetry index

**DOI:** 10.1016/j.mex.2021.101590

**Published:** 2021-11-23

**Authors:** Kai S. Exner

**Affiliations:** a University Duisburg-Essen, Faculty of Chemistry, Theoretical Chemistry, Universitätsstraße 5, 45141 Essen, Germany; b Cluster of Excellence RESOLV, Bochum, Germany; c Center for Nanointegration (CENIDE) Duisburg-Essen, Duisburg, Germany

**Keywords:** Electrocatalysis, Activity prediction, Oxygen evolution reaction, Asymmetric thermodynamic free-energy landscape

## Abstract

The development of oxygen-evolution reaction (OER) electrocatalysts has been spurred by thermodynamic considerations on the free-energy landscape. Most commonly, electrocatalytic activity is approximated by the analysis of the free-energy changes among the mechanistic description, thereby taking only reaction steps with weak-binding adsorbates into account. Herein, a new method, denoted as the electrochemical-step asymmetry index (ESAI), is presented, which approximates electrocatalytic activity by penalizing both too strong as well as too weak bonding of intermediate states in order to mimic the well-known Sabatier principle.

• The electrochemical-step asymmetry index (ESAI) is a descriptor to approximate electrocatalytic activity based on the analysis of the free-energy changes for a given mechanistic description, exemplified by the oxygen evolution reaction (OER).
• The concept of the ESAI is based on the assumption that the optimum free-energy landscape has an asymmetric shape because this may factor overpotential and kinetic effects in the analysis, and the ESAI penalizes both too strong as well as too weak bonding of intermediate states to render a thorough representation of the Sabatier principle feasible.
• The ESAI is a conceptual development of the earlier proposed electrochemical-step symmetry index (ESSI), which relies on a symmetric distribution of the free-energy changes as thermodynamic optimum and which takes only weak-binding adsorbates into account.

## Graphical abstract

**Figure:**
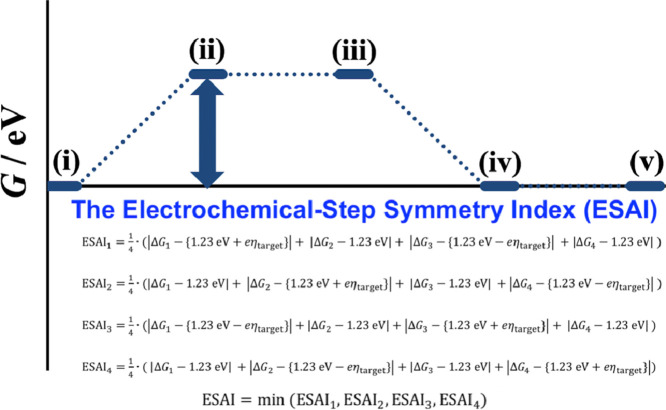

Specifications table

**Table:** 

Subject Area:	Chemistry
More specific subject area:	*Electrocatalysis*
Method name:	*The Electrochemical-Step Asymmetry Index*
Name and reference of original method:	*Electrochemical-Step Symmetry Index (ESSI)* *Reference: Govindarajan, N.; Garcia-Lastra, J. M.; Meijer, E. A.; Calle- Vallejo, F. Does the Breaking of Adsorption-Energy Scaling Relations Guarantee Enhanced Electrocatalysis? Curr. Opin. Electrochem. 2018, 8, 110-117.*
Resource availability:	*Link to:* *Kai S. Exner: Why the Optimum Thermodynamic Free-Energy Landscape of the Oxygen Evolution Reaction Reveals an Asymmetric Shape. Mater. Today Energy 2021, 21, 100831.*

## Method details

The oxygen evolution reaction (OER) is the anodic reaction in electrolyzers to convert water into gaseous oxygen [1]. In computational electrochemistry, the OER is commonly described by four elementary reaction steps (cf. (1), (2), (3)–(4)), which is also denoted as the mononuclear mechanism [2]:

(1) M + H_2_O → M-OH + H^+^ + e^−^ Δ*G*_1_

(2) M-OH → M-O + H^+^ + e^−^ Δ*G*_2_

(3) M-O + H_2_O → M-OOH + H^+^ + e^−^ Δ*G*_3_

(4) M-OOH → M + O_2(g)_ + H^+^ + e^−^ Δ*G*_4_

In (1), (2), (3)–(4), M corresponds to the active site of the electrocatalyst, which is often reconciled with an undercoordinated metal surface atom. The Δ*G*_j_ (j = 1, 2, 3, 4) values indicate the free-energy changes for the respective elementary process.

Recently, Calle-Vallejo and coworkers put forth the electrochemical-step symmetry index (ESSI) as activity descriptor for the OER [3]. The definition of the ESSI is given by Eq. (5):

(5) $$\text{ESSI}=\frac{1}{n}\sum_{j=1}^{n}\left(\frac{\Delta G_{j}^{+}\;-1.23\;\text{eV}}{e}\right)$$

In Equation (5), *e* denotes the elementary charge, and the ∆*G*_j_ values refer to the free-energy changes of (1), (2), (3)–(4). The sum in Equation (5) addresses all free-energy changes ∆*G*_j_^+^ that exceed the equilibrium potential of the OER, *U*^0^_OER_ = 1.23 V *vs*. RHE, on a potential scale; that is, ∆*G*_k_^+^ / *e* > 1.23 V. Here, *n* indicates the number of ∆*G*_j_^+^ values among the mechanistic description of (1), (2), (3)–(4), and therefore, *n* amounts to one, two, or three for the OER. Please note that all free-energy changes ∆*G*_j_^–^ / *e* ≤ 1.23 V are not considered in the analysis [4]. Consequently, the ESSI penalizes all reaction steps in which weak-binding intermediates are formed. In contrast, too strong binding of adsorbates, which can also be detrimental for catalytic activity, is not addressed by the ESSI.

Following the traditional Sabatier principle, the binding of an adsorbate to a catalyst surface should be neither to strong nor too weak [5]. As a consequence, it would be desirable to derive an activity descriptor that follows the idea of the Sabatier principle by penalizing both too strong and too weak bonding of intermediates for the approximation of electrocatalytic activity [6].

While the ESSI corresponds to the picture of a symmetric free-energy diagram as thermodynamic ideal (all Δ*G*_j_ (j = 1, 2, 3, 4) values are equal to 1.23 eV), the symmetric picture does not capture overpotential and kinetic effects in the analysis [7]. Recently, it was demonstrated for a two-electron process, such as the hydrogen evolution reaction, that the optimum thermodynamic free-energy surface changes from a symmetric to an asymmetric landscape with increasing overpotential. [7,8] Therefore, to derive an improved activity measure compared to the ESSI, it is not only required to penalize both too strong and too weak bonding of intermediates, but also to use the concept of the asymmetric free-energy landscape as thermodynamic optimum because this may factor the applied overpotential and the reaction kinetic in the analysis of adsorption free energies [6,9]. These facts give rise to the introduction of the electrochemical-step asymmetry (ESAI).

The idea of the ESAI is as follows: given that the OER is described by four proton-electron coupled transfer steps, there are four potentially rate-determining steps (RDS referring to the kinetics) in the high overpotential regime where the Tafel slope exceeds 60 mV/dec.: either OH adsorption (cf. Equation (1)), O formation (cf. Equation (2)), OOH formation (cf. Equation (3)), or OOH decomposition (cf. Equation (4)). The thermodynamics (Δ*G*_j_ value) of the respective RDS (OH adsorption, O formation, OOH formation, or OOH decomposition) is thought to be thermoneutral at the chosen target overpotential [6,7]. In order to achieve thermoneutral bonding of the key adsorbate at the target overpotential, the energetics of the Δ*G*_j_ (j = 1, 2, 3, 4) values is corrected by the term *eη*_target_ for the key adsorbate; e. g., for OH adsorption, the Δ*G*_1_ value has to be adjusted by + *eη*_target_. To meet the criterion that the Δ*G*_j_ (j = 1, 2, 3, 4) values sum up to 4.92 eV, the Δ*G*_3_ value is lowered by *eη*_target_. In the same fashion, for O formation the Δ*G*_2_ value is increased by the term *eη*_target_ to render thermoneutral bonding of the O adsorbate at the target overpotential feasible; thus, the Δ*G*_4_ value is lowered *eη*_target_. Altogether, the following conclusions for the free-energy changes of the elementary reaction steps can be made:

a) *RDS OH adsorption*: Δ*G*_1_ = 1.23 eV + *eη*_target_, Δ*G*_2_ = 1.23 eV, Δ*G*_3_ = 1.23 eV – *eη*_target_, Δ*G*_4_ = 1.23 eV
b) *RDS O formation*: Δ*G*_1_ = 1.23 eV, Δ*G*_2_ = 1.23 eV + *eη*_target_, Δ*G*_3_ = 1.23 eV, Δ*G*_4_ = 1.23 eV – *eη*_target_
c) *RDS OOH formation*: Δ*G*_1_ = 1.23 eV – *eη*_target_, Δ*G*_2_ = 1.23 eV, Δ*G*_3_ = 1.23 eV + *eη*_target_, Δ*G*_4_ = 1.23 eV
d) *RDS OOH decomp.*: Δ*G*_1_ = 1.23 eV, Δ*G*_2_ = 1.23 eV – *eη*_target_, Δ*G*_3_ = 1.23 eV, Δ*G*_4_ = 1.23 eV + *eη*_target_

Consequently, we can define four ESAI_j_ (j = 1, 2, 3, 4) values, in which in each case a different reaction step is thought to be the RDS. Herein, in each part of the sum the deviation of the actual free-energy change from its optimum value according to the asymmetric free-energy landscape as reference is evaluated (cf. (6), (7), (8)–9)):

(6) $$\text{ESA}I_{1}=\frac{1}{4}\cdot \left(\left|\Delta G_{1}-\left\{1.23\text{eV}+e\eta_{\text{target}}\right\}\right|+\left|\Delta G_{2}-1.23\;\text{eV}\right|+\left|\Delta G_{3}-\left\{1.23\;\text{eV}-e\eta_{\text{target}}\right\}\right|+\left|\Delta G_{4}-1.23\;\text{eV}\right|\right)$$

(7) $$\text{ESA}I_{2}=\frac{1}{4}\cdot \left(\left|\Delta G_{1}-1.23\;\text{eV}\right|+\left|\Delta G_{2}-\left\{1.23\;\text{eV}+e\eta_{\text{target}}\right\}\right|+\left|\Delta G_{3}-1.23\;\text{eV}\right|+\left|\Delta G_{4}-\left\{1.23\;\text{eV}-e\eta_{\text{target}}\right\}\right|\right)$$

(8) $$\text{ESA}I_{3}=\frac{1}{4}\cdot \left(\left|\Delta G_{1}-\left\{1.23\;\text{eV}-e\eta_{\text{target}}\right\}\right|+\left|\Delta G_{2}-1.23\;\text{eV}\right|+\left|\Delta G_{3}-\left\{1.23\;\text{eV}+e\eta_{\text{target}}\right\}\right|+\left|\Delta G_{4}-1.23\;\text{eV}\right|\right)$$

(9) $$\text{ESA}I_{4}=\frac{1}{4}\cdot \left(\left|\Delta G_{1}-1.23\;\text{eV}\right|+\left|\Delta G_{2}-\left\{1.23\;\text{eV}-e\eta_{\text{target}}\right\}\right|+\left|\Delta G_{3}-1.23\;\text{eV}\right|+\left|\Delta G_{4}-\left\{1.23\;\text{eV}+e\eta_{\text{target}}\right\}\right|\right)$$

Considering that the OER mechanism is analyzed by thermodynamic considerations only, we do not know a priori for an electrocatalyst which of the four steps (OH adsorption, O formation, OOH formation, or OOH decomposition) refers to the limiting process [10]. Yet, we can approximate the RDS by assuming that the ESAI for the limiting step should be the smallest among the set of the ESAI_j_ (j = 1, 2, 3, 4) values because this situation is most likely observed at the target overpotential when taking the Sabatier principle as well as the Brønsted−Evans−Polanyi relation into account [6]. Therefore, we can define the ESAI as the minimum of the four scenarios referring to (6), (7), (8)–9):

(10) $$\text{ESAI}=\text{min}\left(\text{ESA}I_{1},\;\text{ESA}I_{2},\text{ESA}I_{3},\text{ESA}I_{4}\right)$$

The ESAI, as given by Equation (10), is an activity descriptor for a four-electron process, such as the oxygen evolution and oxygen reduction reactions. I would like to emphasize, though, that the concept of the ESAI can also be transferred to any other multiple-electron process in electrocatalysis to render activity predictions based on the extended Sabatier principle feasible [9,10]. This can be achieved by the following procedure (cf. (11), (12), (13), (14)–15)):

(11) $$\text{ESAI}=\min\left(\text{ESA}I_{k}\right),\;k=1,\;\ldots ,\;n$$

(12) $$\text{ESA}I_{1}=\frac{1}{n}\left(\left|\Delta G_{1}-\left\{eU_{\text{eq}}+e\eta_{\text{target}}\right\}\right|+\left|\Delta G_{3}-\left\{eU_{\text{eq}}-e\eta_{\text{target}}\right\}\right|+\sum_{j=1}^{n}\left|\Delta G_{j\neq 1,j\neq 3}-eU_{\text{eq}}\right|\right)$$

(13) $$\text{ESA}I_{2}=\frac{1}{n}\left(\left|\Delta G_{2}-\left\{eU_{\text{eq}}+e\eta_{\text{target}}\right\}\right|+\left|\Delta G_{4}-\left\{eU_{\text{eq}}-e\eta_{\text{target}}\right\}\right|+\sum_{j=1}^{n}\left|\Delta G_{j\neq 2,j\neq 4}-eU_{\text{eq}}\right|\right)$$

(14) $$\text{ESA}I_{k}=\frac{1}{n}\left(\left|\Delta G_{k}-\left\{eU_{\text{eq}}+e\eta_{\text{target}}\right\}\right|+\left|\Delta G_{k+2}-\left\{eU_{\text{eq}}-e\eta_{\text{target}}\right\}\right|+\sum_{j=1}^{n}\left|\Delta G_{j\neq k,j\neq k+2}-eU_{\text{eq}}\right|\right)$$

(15) $$\text{ESA}I_{n}=\frac{1}{n}\left(\left|\Delta G_{n}-\left\{eU_{\text{eq}}+e\eta_{\text{target}}\right\}\right|+\left|\Delta G_{2}-\left\{eU_{\text{eq}}-e\eta_{\text{target}}\right\}\right|+\sum_{j=1}^{n}\left|\Delta G_{j\neq n,j\neq 2}-eU_{\text{eq}}\right|\right)$$

In (11), (12), (13), (14)–15), *U*_eq_ refers to the equilibrium potential of the respective electrocatalytic process, in which *n* electron-transfer steps take place. As a consequence, *n* different ESAI_k_ values can be specified in that the optimum free-energy change for the key adsorbate is adjusted by the term *eη*_target_ to meet thermoneutral bonding at the target overpotential. In turn, this causes that the optimum free-energy change for the second adsorbate following the key intermediate is lowered by *eη*_target_. The ESAI is then given by the smallest ESAI_k_ value to establish a connection of the key adsorbate to the kinetics in terms of the RDS. An in-depth discussion relating to the application of the ESAI can be found elsewhere [6], given that therein the ESAI is exemplified by the OER over transition-metal oxides, metal oxides, perovskites, functionalized graphitic materials, and porphyrins. The present methodological contribution, however, illustrates the reasoning of how to translate the concept of the ESSI to the ESAI by the consideration of overpotential and kinetic effects as well as correct application of the Sabatier principle by penalizing both too strong and too weak bonding of reaction intermediates.

Supplementary material and/or Additional information:

## Declaration of Competing Interest

The authors declare that they have no known competing financial interests or personal relationships that could have appeared to influence the work reported in this paper.

## Appendix Supplementary materials

Image, application 1
