# Supplementary material for: The electrochemical-step asymmetry index
Source: MethodsX. 2021 Nov 23;8:101590. doi: 10.1016/j.mex.2021.101590 (PMC8720886; doi:10.1016/j.mex.2021.101590)
Supplement: Supplementary file 1 [file mmc1.pdf]

Methods article template for submitting to MethodsX

- *Please fill in the template below and delete all instructional text in italics*
- *If you have any questions, please contact the journal at mexjm@elsevier.com*

Method Article – Title Page

**Title**	*The Electrochemical-Step Asymmetry Index*
**Authors**	*Kai S. Exner[1,2,3,*]*
**Affiliations**	*[1] University Duisburg-Essen, Faculty of Chemistry, Theoretical Chemistry, Universitätsstraße 5, 45141 Essen, Germany*<br>*[2] Cluster of Excellence RESOLV, Bochum, Germany*<br>*[3] Center for Nanointegration (CENIDE) Duisburg-Essen, Duisburg, Germany*
**Corresponding Author's email address**	*kai.exner@uni-due.de*
**Keywords**	• *electrocatalysis*<br>• *activity prediction*<br>• *oxygen evolution reaction*<br>• *asymmetric thermodynamic free-energy landscape*
**Direct Submission or Co-Submission**<br><br>*Co-submissions are papers that have been submitted alongside an original research paper accepted for publication by another Elsevier journal*	*Please select*<br>*Co-Submission*<br>*Kai S. Exner: Why the Optimum Thermodynamic Free-Energy Landscape of the Oxygen Evolution Reaction Reveals an Asymmetric Shape. Mater. Today Energy 2021, 21, 100831.*

ABSTRACT

The development of oxygen-evolution reaction (OER) electrocatalysts has been spurred by thermodynamic considerations on the free-energy landscape. Most commonly, electrocatalytic activity is approximated by the analysis of the free-energy changes among the mechanistic description, thereby taking only reaction steps with weak-binding adsorbates into account. Herein, a new method, denoted as the electrochemical-step asymmetry index (ESAI), is presented, which approximates electrocatalytic activity by penalizing both too strong as well as too weak bonding of intermediate states in order to mimic the well-known Sabatier principle.

- *The electrochemical-step asymmetry index (ESAI) is a descriptor to approximate electrocatalytic activity based on the analysis of the free-energy changes for a given mechanistic description, exemplified by the oxygen evolution reaction (OER).*
- *The concept of the ESAI is based on the assumption that the optimum free-energy landscape has an asymmetric shape because this may factor overpotential and kinetic effects in the analysis, and the ESAI penalizes both too strong as well as too weak bonding of intermediate states to render a thorough representation of the Sabatier principle feasible.*
- *The ESAI is a conceptual development of the earlier proposed electrochemical-step symmetry index (ESSI), which relies on a symmetric distribution of the free-energy changes as thermodynamic optimum and which takes only weak-binding adsorbates into account.*

SPECIFICATIONS TABLE

**Subject Area**	Chemistry
**More specific subject area**	*Electrocatalysis*
**Method name**	*The Electrochemical-Step Asymmetry Index*
**Name and reference of original method**	*Electrochemical-Step Symmetry Index (ESSI)*<br><br>*Reference: Govindarajan, N.; Garcia-Lastra, J. M.; Meijer, E. A.; Calle- Vallejo, F. Does the Breaking of Adsorption-Energy Scaling Relations Guarantee Enhanced Electrocatalysis? Curr. Opin. Electrochem. 2018, 8, 110-117.*
**Resource availability**	*Link to:*

	Kai S. Exner: *Why the Optimum Thermodynamic Free-Energy Landscape of the Oxygen Evolution Reaction Reveals an Asymmetric Shape. Mater. Today Energy 2021, 21, 100831.*

***Method details**

The oxygen evolution reaction (OER) is the anodic reaction in electrolyzers to convert water into gaseous oxygen.[1] In computational electrochemistry, the OER is commonly described by four elementary reaction steps (cf. equations (1) – (4)), which is also denoted as the mononuclear mechanism:[2]

M + H_2O	→ M-OH + H^+ + e^-	ΔG_1	(1)
M-OH	→ M-O + H^+ + e^-	ΔG_2	(2)
M-O + H_2O	→ M-OOH + H^+ + e^-	ΔG_3	(3)
M-OOH	→ M + $O_{2(g)}$ + H^+ + e^-	ΔG_4	(4)

In equations (1) – (4), M corresponds to the active site of the electrocatalyst, which is often reconciled with an undercoordinated metal surface atom. The ΔG_j (j = 1, 2, 3, 4) values indicate the free-energy changes for the respective elementary process.

Recently, Calle-Vallejo and coworkers put forth the electrochemical-step symmetry index (ESSI) as activity descriptor for the OER.[3] The definition of the ESSI is given by equation (5):

$$\mathrm{ESSI} = \frac{1}{n}\sum_{j=1}^{n}\left(\frac{\Delta G_j^{+} - 1.23\ \mathrm{eV}}{e}\right) \qquad (5)$$

In equation (5), *e* denotes the elementary charge, and the ΔG_j values refer to the free-energy changes of equations (1) – (4). The sum in equation (5) addresses all free-energy changes ΔG_j^+ that exceed the equilibrium potential of the OER, U^0_{OER} = 1.23 V *vs.* RHE, on a potential scale; that is, $\Delta G_k^+ / e > 1.23$ V. Here, *n* indicates the number of ΔG_j^+ values among the mechanistic description of equations (1) – (4), and therefore, *n* amounts to one, two, or three for the OER. Please note that all free-energy changes $\Delta G_j^- / e \leq 1.23$ V are not considered in the analysis.[4] Consequently, the ESSI penalizes all reaction steps in which weak-binding intermediates are formed. In contrast, too strong binding of adsorbates, which can also be detrimental for catalytic activity, is not addressed by the ESSI.

Following the traditional Sabatier principle, the binding of an adsorbate to a catalyst surface should be neither to strong nor too weak.[5] As a consequence, it would be desirable to derive an activity descriptor that follows the idea of the Sabatier principle by penalizing both too strong and too weak bonding of intermediates for the approximation of electrocatalytic activity.[6]

While the ESSI corresponds to the picture of a symmetric free-energy diagram as thermodynamic ideal (all ΔG_j (j = 1, 2, 3, 4) values are equal to 1.23 eV), the symmetric picture does not capture overpotential and kinetic effects in the analysis.[7] Recently, it was demonstrated for a two-electron process, such as the hydrogen evolution reaction, that the optimum thermodynamic free-energy surface changes from a symmetric to an asymmetric landscape with increasing overpotential.[7,8] Therefore, to derive an improved activity measure compared to the ESSI, it is not only required to penalize both too strong and too weak bonding of intermediates, but also to use the concept of the asymmetric free-energy landscape as thermodynamic optimum because this may factor the applied overpotential and the reaction kinetic in the analysis of adsorption free energies.[6,9] These facts give rise to the introduction of the electrochemical-step asymmetry (ESAI).

The idea of the ESAI is as follows: given that the OER is described by four proton-electron coupled transfer steps, there are four potentially rate-determining steps (RDS referring to the kinetics) in the high overpotential regime where the Tafel slope exceeds 60 mV/dec.: either OH adsorption (cf. equation (1)), O formation (cf. equation (2)), OOH formation (cf. equation (3)), or OOH decomposition (cf. equation (4)). The thermodynamics (ΔG_j value) of the respective RDS (OH adsorption, O formation, OOH formation, or OOH decomposition) is thought to be thermoneutral at the chosen target overpotential.[6,7] In order to achieve thermoneutral bonding of the key adsorbate at the target overpotential, the energetics of the ΔG_j (j = 1, 2, 3, 4) values is corrected by the term $e\eta_{target}$ for the key adsorbate; e. g., for OH adsorption, the ΔG_1 value has to be adjusted by + $e\eta_{target}$. To meet the criterion that

the ΔG_j (j = 1, 2, 3, 4) values sum up to 4.92 eV, the ΔG_3 value is lowered by $e\eta_{target}$. In the same fashion, for O formation the ΔG_2 value is increased by the term $e\eta_{target}$ to render thermoneutral bonding of the O adsorbate at the target overpotential feasible; thus, the ΔG_4 value is lowered $e\eta_{target}$. Altogether, the following conclusions for the free-energy changes of the elementary reaction steps can be made:

a) RDS OH adsorption: ΔG_1 = 1.23 eV + $e\eta_{target}$, ΔG_2 = 1.23 eV, ΔG_3 = 1.23 eV – $e\eta_{target}$, ΔG_4 = 1.23 eV

b) RDS O formation: ΔG_1 = 1.23 eV, ΔG_2 = 1.23 eV + $e\eta_{target}$, ΔG_3 = 1.23 eV, ΔG_4 = 1.23 eV – $e\eta_{target}$

c) RDS OOH formation: ΔG_1 = 1.23 eV – $e\eta_{target}$, ΔG_2 = 1.23 eV, ΔG_3 = 1.23 eV + $e\eta_{target}$, ΔG_4 = 1.23 eV

d) RDS OOH decomp.: ΔG_1 = 1.23 eV, ΔG_2 = 1.23 eV – $e\eta_{target}$, ΔG_3 = 1.23 eV, ΔG_4 = 1.23 eV + $e\eta_{target}$

Consequently, we can define four ESAI_j (j = 1, 2, 3, 4) values, in which in each case a different reaction step is thought to be the RDS. Herein, in each part of the sum the deviation of the actual free-energy change from its optimum value according to the asymmetric free-energy landscape as reference is evaluated (cf. equations (6) – (9)):

$$\text{ESAI}_1 = \frac{1}{4}\cdot\left(\left|\Delta G_1 - \{1.23\text{ eV} + e\eta_{\text{target}}\}\right| + \left|\Delta G_2 - 1.23\text{ eV}\right| + \left|\Delta G_3 - \{1.23\text{ eV} - e\eta_{\text{target}}\}\right| + \left|\Delta G_4 - 1.23\text{ eV}\right|\right) \quad (6)$$

$$\text{ESAI}_2 = \frac{1}{4}\cdot\left(\left|\Delta G_1 - 1.23\text{ eV}\right| + \left|\Delta G_2 - \{1.23\text{ eV} + e\eta_{\text{target}}\}\right| + \left|\Delta G_3 - 1.23\text{ eV}\right| + \left|\Delta G_4 - \{1.23\text{ eV} - e\eta_{\text{target}}\}\right|\right) \quad (7)$$

$$\text{ESAI}_3 = \frac{1}{4}\cdot\left(\left|\Delta G_1 - \{1.23\text{ eV} - e\eta_{\text{target}}\}\right| + \left|\Delta G_2 - 1.23\text{ eV}\right| + \left|\Delta G_3 - \{1.23\text{ eV} + e\eta_{\text{target}}\}\right| + \left|\Delta G_4 - 1.23\text{ eV}\right|\right) \quad (8)$$

$$\text{ESAI}_4 = \frac{1}{4}\cdot\left(\left|\Delta G_1 - 1.23\text{ eV}\right| + \left|\Delta G_2 - \{1.23\text{ eV} - e\eta_{\text{target}}\}\right| + \left|\Delta G_3 - 1.23\text{ eV}\right| + \left|\Delta G_4 - \{1.23\text{ eV} + e\eta_{\text{target}}\}\right|\right) \quad (9)$$

Considering that the OER mechanism is analyzed by thermodynamic considerations only, we do not know a priori for an electrocatalyst which of the four steps (OH adsorption, O formation, OOH formation, or OOH decomposition) refers to the limiting process.[10] Yet, we can approximate the RDS by assuming that the ESAI for the limiting step should be the smallest among the set of the ESAI_j (j = 1, 2, 3, 4) values because this situation is most likely observed at the target overpotential when taking the Sabatier principle as well as the Brønsted−Evans−Polanyi relation into account.[6] Therefore, we can define the ESAI as the minimum of the four scenarios referring to equations (6) – (9):

$$\text{ESAI} = \min\left(\text{ESAI}_1, \text{ESAI}_2, \text{ESAI}_3, \text{ESAI}_4\right) \quad (10)$$

The ESAI, as given by equation (10), is an activity descriptor for a four-electron process, such as the oxygen evolution or reduction reactions. I would like to emphasize, though, that the concept of the ESAI can also be transferred to any other multiple-electron process in electrocatalysis to render activity predictions based on the extended Sabatier principle.[9,10] This can be achieved by the following procedure (cf. equations (11) – (15)):

$$\text{ESAI} = \min\left(\text{ESAI}_k\right), k = 1, \dots, n \quad (11)$$

$$\text{ESAI}_1 = \frac{1}{n}\left(\left|\Delta G_1 - \{eU_{\text{eq}} + e\eta_{\text{target}}\}\right| + \left|\Delta G_3 - \{eU_{\text{eq}} - e\eta_{\text{target}}\}\right| + \textstyle\sum_{j=1}^{n}\left|\Delta G_{j\neq 1, j\neq 3} - eU_{\text{eq}}\right|\right) \quad (12)$$

$$\text{ESAI}_2 = \frac{1}{n}\left(\left|\Delta G_2 - \{eU_{\text{eq}} + e\eta_{\text{target}}\}\right| + \left|\Delta G_4 - \{eU_{\text{eq}} - e\eta_{\text{target}}\}\right| + \textstyle\sum_{j=1}^{n}\left|\Delta G_{j\neq 2, j\neq 4} - eU_{\text{eq}}\right|\right) \quad (13)$$

…

$$\text{ESAI}_k = \frac{1}{n}\left(\left|\Delta G_k - \{eU_{\text{eq}} + e\eta_{\text{target}}\}\right| + \left|\Delta G_{k+2} - \{eU_{\text{eq}} - e\eta_{\text{target}}\}\right| + \textstyle\sum_{j=1}^{n}\left|\Delta G_{j\neq k, j\neq k+2} - eU_{\text{eq}}\right|\right) \quad (14)$$

…

$$\text{ESAI}_n = \frac{1}{n}\left(\left|\Delta G_n - \{eU_{\text{eq}} + e\eta_{\text{target}}\}\right| + \left|\Delta G_2 - \{eU_{\text{eq}} - e\eta_{\text{target}}\}\right| + \textstyle\sum_{j=1}^{n}\left|\Delta G_{j\neq n, j\neq 2} - eU_{\text{eq}}\right|\right) \quad (15)$$

In equations (11) – (15), U_{eq} refers to the equilibrium potential of the respective electrocatalytic process, in which n electron-transfer steps take place. As a consequence, n different $ESAI_k$ values can be specified in that the optimum free-energy change for the key adsorbate is adjusted by the term $e\eta_{target}$ to meet thermoneutral bonding at the target overpotential. In turn, this causes that the optimum free-energy change for the second adsorbate following the key intermediate is lowered by $e\eta_{target}$. The ESAI is then given by the smallest $ESAI_k$ value to render the connection of the key adsorbate to the kinetics in terms of the RDS feasible. An in-depth discussion relating to the application of the ESAI can be found elsewhere,[6] given that therein the ESAI is exemplified by the OER over transition-metal oxides, metal oxides, perovskites, functionalized graphitic materials, and porphyrins. The present methodological contribution, however, illustrates the reasoning of how to translate the concept of the ESSI to the ESAI by the consideration of overpotential and kinetic effects as well as correct application of the Sabatier principle by penalizing both too strong and too weak bonding of reaction intermediates.

Acknowledgements:
KSE acknowledges funding by the Ministry of Culture and Science of the Federal State of North Rhine-Westphalia (NRW Return Grant). KSE is associated with the CRC/TRR247: "Heterogeneous Oxidation Catalysis in the Liquid Phase" (Project number 388390466-TRR 247) and the Center for Nanointegration (CENIDE). This article is based upon the work from COST Action 18234, supported by COST (European Cooperation in Science and Technology). Funded by the Deutsche Forschungsgemeinschaft (DFG, German Research Foundation) under Germany's Excellence Strategy - EXC-2033 – 390677874 - RESOLV.

Declaration of interests:

X The authors declare that they have no known competing financial interests or personal relationships that could have appeared to influence the work reported in this paper.

☐ The authors declare the following financial interests/personal relationships which may be considered as potential competing interests:

Supplementary material *and/or* Additional information: -

***References:**

[1] Masa, J.; Andronescu, C.; Schuhmann, W. Electrocatalysis as the Nexus for Sustainable Renewable Energy: The Gordian Knot of Activity, Stability, and Selectivity. *Angew. Chem. Int. Ed.* **2020**, *59*, 15298-15312.

[2] Rossmeisl, J.; Qu, Z.-W.; Zhu, H.; Kroes, G.-J.; Nørskov, J. K. Electrolysis of Water on Oxide Surfaces. *J. Electroanal. Chem.* **2007**, *607*, 83-89.

[3] Govindarajan, N.; Garcia-Lastra, J. M.; Meijer, E. A.; Calle- Vallejo, F. Does the Breaking of Adsorption-Energy Scaling Relations Guarantee Enhanced Electrocatalysis? *Curr. Opin. Electrochem.* **2018**, *8*, 110-117.

[4] Pique, O.; Illas, F.; Calle-Vallejo, F. Designing Water Splitting Catalysts Using Rules of Thumb: Advantages, Dangers and Alternatives. *Phys. Chem. Chem. Phys.* **2020**, *22*, 6797-6803.

[5] Che, M. Nobel Prize in chemistry 1912 to Sabatier: Organic chemistry or catalysis? *Catal. Tod.* **2013**, *218*, 162-171.

[6] Exner, K. S. Why the Optimum Thermodynamic Free-Energy Landscape of the Oxygen Evolution Reaction Reveals an Asymmetric Shape. *Mater. Today Energy* **2021**, *21*,100831.

[7] Exner, K. S. Does a Thermoneutral Electrocatalyst Correspond to the Apex of a Volcano Plot for a Simple Two-Electron Process? *Angew. Chem. Int. Ed.* **2020**, *59*, 10236–10240.

[8] Ooka, H.; Wintzer, M.E.; Nakamura, R. Non-Zero Binding Enhances Kinetics of Catalysis: Machine Learning Analysis on the Experimental Hydrogen Binding Energy of Platinum. *ACS Catal.* **2021**, *11*, 6298-6303.

[9] Exner, K. S. A Universal Descriptor for the Screening of Electrode Materials for Multiple-Electron Processes: Beyond the Thermodynamic Overpotential, *ACS Catal.* **2020**, *10*, 12607-12617.

[10] Exner, K. S. Why approximating electrocatalytic activity by a single free-energy change is insufficient. *Electrochim. Acta* **2021**, *375*, 137975.

Graphical Abstract

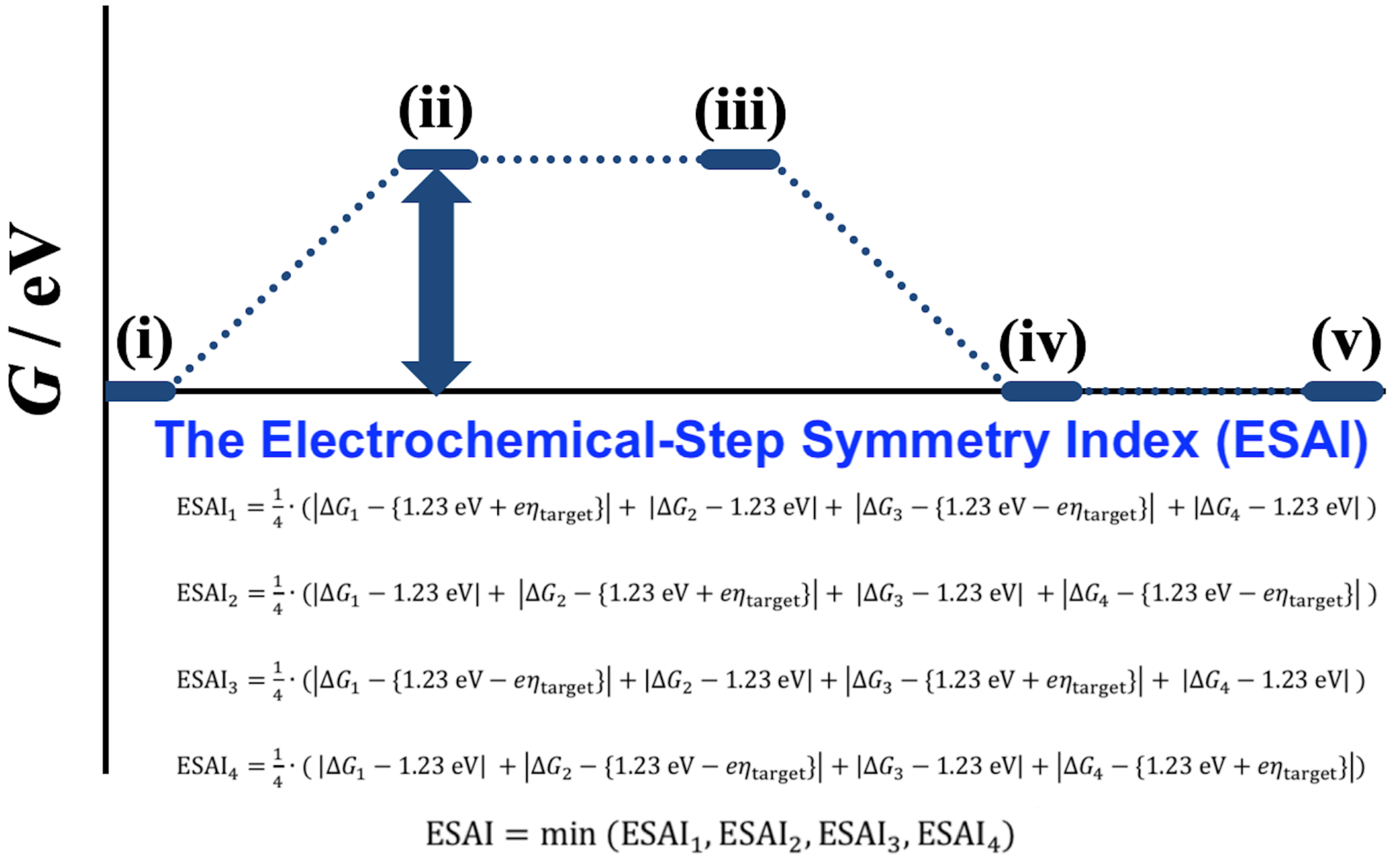

The Electrochemical-Step Symmetry Index (ESAI)

$$\mathrm{ESAI}_1 = \frac{1}{4} \cdot \left(\left|\Delta G_1 - \{1.23\ \mathrm{eV} + e\eta_{\mathrm{target}}\}\right| + \left|\Delta G_2 - 1.23\ \mathrm{eV}\right| + \left|\Delta G_3 - \{1.23\ \mathrm{eV} - e\eta_{\mathrm{target}}\}\right| + \left|\Delta G_4 - 1.23\ \mathrm{eV}\right| \right)$$

$$\mathrm{ESAI}_2 = \frac{1}{4} \cdot \left(\left|\Delta G_1 - 1.23\ \mathrm{eV}\right| + \left|\Delta G_2 - \{1.23\ \mathrm{eV} + e\eta_{\mathrm{target}}\}\right| + \left|\Delta G_3 - 1.23\ \mathrm{eV}\right| + \left|\Delta G_4 - \{1.23\ \mathrm{eV} - e\eta_{\mathrm{target}}\}\right| \right)$$

$$\mathrm{ESAI}_3 = \frac{1}{4} \cdot \left(\left|\Delta G_1 - \{1.23\ \mathrm{eV} - e\eta_{\mathrm{target}}\}\right| + \left|\Delta G_2 - 1.23\ \mathrm{eV}\right| + \left|\Delta G_3 - \{1.23\ \mathrm{eV} + e\eta_{\mathrm{target}}\}\right| + \left|\Delta G_4 - 1.23\ \mathrm{eV}\right| \right)$$

$$\mathrm{ESAI}_4 = \frac{1}{4} \cdot \left(\left|\Delta G_1 - 1.23\ \mathrm{eV}\right| + \left|\Delta G_2 - \{1.23\ \mathrm{eV} - e\eta_{\mathrm{target}}\}\right| + \left|\Delta G_3 - 1.23\ \mathrm{eV}\right| + \left|\Delta G_4 - \{1.23\ \mathrm{eV} + e\eta_{\mathrm{target}}\}\right| \right)$$

$$\mathrm{ESAI} = \min\left(\mathrm{ESAI}_1, \mathrm{ESAI}_2, \mathrm{ESAI}_3, \mathrm{ESAI}_4\right)$$
